# Supplementary material for: Tissue-specific RNA-seq defines genes governing male tail tip morphogenesis in C. elegans
Source: bioRxiv. 2024 Jan 12:2024.01.12.575210. Preprint. [Version 1] doi: 10.1101/2024.01.12.575210 (PMC10802606; doi:10.1101/2024.01.12.575210)
Supplement: Supplement 2 [file media-2.pdf]

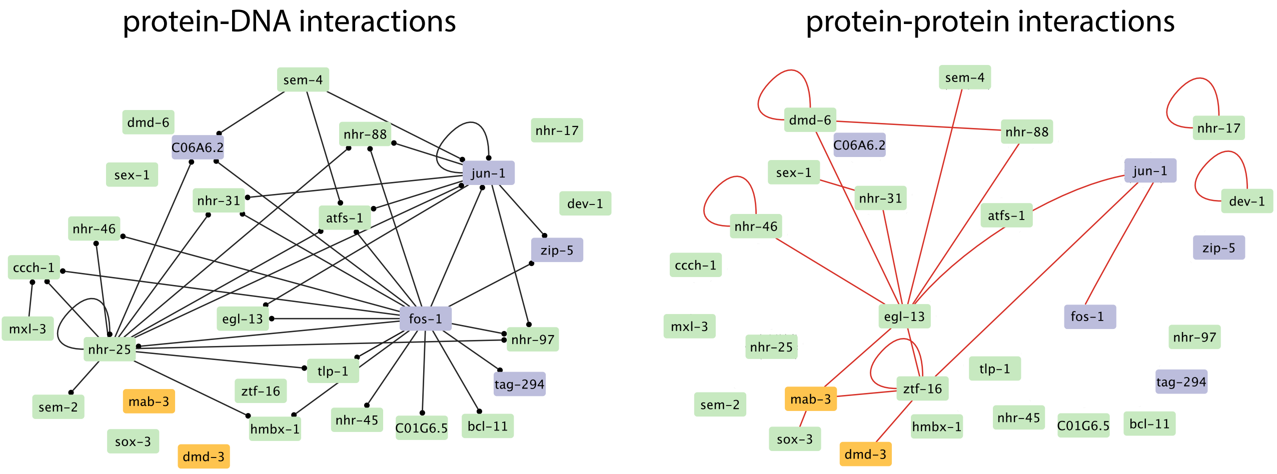

Figure S2
TF-target interactions (from TFlink) and protein-protein interactions (Reece-Hoyes et al., 2013) between TFs that are regulated by DMD-3 (DMD-3-activated genes in purple, DMD-3-repressed genes in green).
