# Supplementary material for: Tissue-specific RNA-seq defines genes governing male tail tip morphogenesis in C. elegans
Source: bioRxiv. 2024 Jan 12:2024.01.12.575210. Preprint. [Version 1] doi: 10.1101/2024.01.12.575210 (PMC10802606; doi:10.1101/2024.01.12.575210)
Supplement: Supplement 3 [file media-3.pdf]

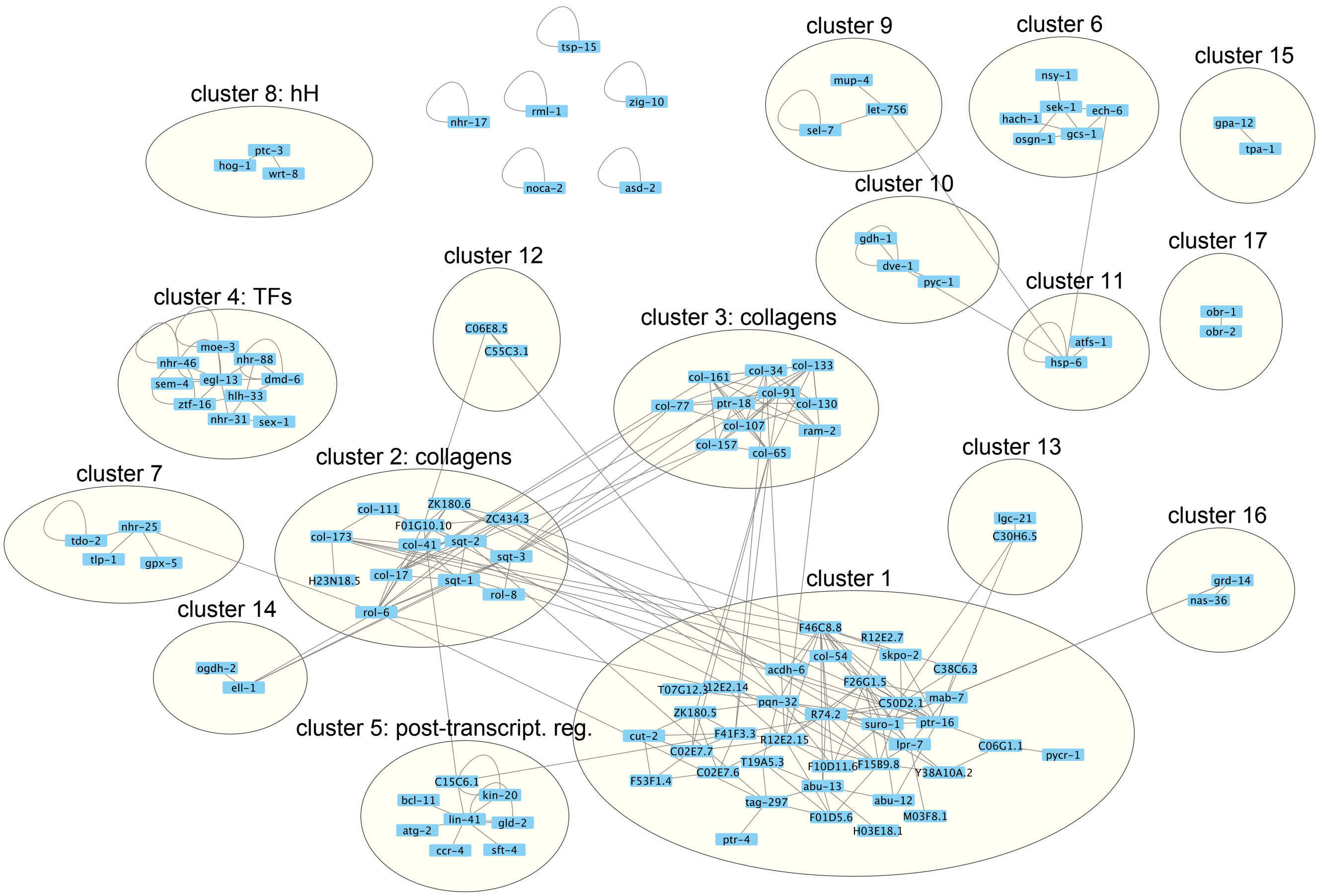


**Figure S3**

Clustering of genes repressed by DMD-3 by interactions mined from WormBase. Interaction cluster 1 contains 20 genes of unknown function.
